# Assessment of human resources management practices in Lebanese hospitals

**DOI:** 10.1186/1478-4491-7-84

**Published:** 2009-11-12

**Authors:** Fadi El-Jardali, Victoria Tchaghchagian, Diana Jamal

**Affiliations:** 1 Health Management and Policy Department, Faculty of Health Sciences, American University of Beirut, Beirut, Lebanon

## Abstract

**Background:**

Sound human resources (HR) management practices are essential for retaining effective professionals in hospitals. Given the recruitment and retention reality of health workers in the twenty-first century, the role of HR managers in hospitals and those who combine the role of HR managers with other responsibilities should not be underestimated. The objective of this study is to assess the perception of HR managers about the challenges they face and the current strategies being adopted. The study also aims at assessing enabling factors including role, education, experience and HR training.

**Methods:**

A cross-sectional survey design of HR managers (and those who combine their role as HR manager with other duties) in Lebanese hospitals was utilized. The survey included a combination of open- and close-ended questions. Questions included educational background, work experience, and demographics, in addition to questions about perceived challenges and key strategies being used. Quantitative data analysis included uni-variate analysis, whereas thematic analysis was used for open-ended questions.

**Results:**

A total of 96 respondents from 61 hospitals responded. Respondents had varying levels of expertise in the realm of HR management. Thematic analysis revealed that challenges varied across respondents and participating hospitals. The most frequently reported challenge was poor employee retention (56.7%), lack of qualified personnel (35.1%), and lack of a system for performance evaluation (28.9%). Some of the strategies used to mitigate the above challenges included offering continuing education and training for employees (19.6%), improving salaries (14.4%), and developing retention strategies (10.3%). Mismatch between reported challenges and strategies were observed.

**Conclusion:**

To enable hospitals to deliver good quality, safe healthcare, improving HR management is critical. There is a need for a cadre of competent HR managers who can fully assume these responsibilities and who can continuously improve the status of employees at their organizations. The upcoming accreditation survey of Lebanese hospitals (2010-2011) presents an opportunity to strengthen HR management and enhance competencies of existing HR managers. Recognizing HR challenges and the importance of effective HR strategies should become a priority to policy makers and top managers alike. Study findings may extend to other countries in the Eastern Mediterranean region.

## Background

The 2006 World Health Report [1] launched the Health Workforce Decade (2006-2015), with high priority given to retaining high-quality health care workers. The Kampala Declaration (2008) stressed the crucial role of retaining an effective, responsive and equitably distributed health workforce [2]. Sound human resources (HR) management practices are a key strategy for retaining effective health professionals in health care organizations (HCOs). Given the recruitment and retention reality of the health workforce in the twenty-first century, the role of HR managers in health care organizations (HCOs) and those who combine the role of HR managers with other responsibilities should not be underestimated.

One of the biggest challenges for hospitals today is the availability of a strong, capable, and motivated workforce. Hospitals are 'people-driven' and their primary expenses are labour costs. As in many developed and developing countries, many hospitals in Middle Eastern countries have come to realize that the most important asset to their organization, besides physical capital and consumables, is their health human resources, without which they cannot properly function [3]. At the system level, evidence indicates a strong link between the availability of health care providers and population health outcomes [4].

Poor work environments and the absence of sound recruitment and retention practices are some of the key health human resources challenges that are facing many Middle Eastern hospitals. These obstacles have resulted in growing staff shortages, attrition and early retirement, poor staff satisfaction, high turnover, and emigration [5]. Many hospitals suffer from poor managerial and planning capacity in the area of health human resources, and lack recruitment and retention strategies. Such strategies are essential in terms of planning, job satisfaction, and intent to stay [6]. Few studies have been conducted to assess recruitment and retention practices and strategies in the Eastern Mediterranean Region (EMR). A study targeting nursing directors in Lebanon found that the majority of the sampled hospitals (88.2%) reported facing challenges in retaining their nurses due to unsatisfactory salary and benefits (80.8%); unsuitable shifts and working hours (38.4%); presence of better opportunities abroad (30.1%) and within the country (30.1%); workload (27.4%); and instability of the country (16.4%) [6]. Many hospitals reported engaging in strategies to mitigate the above challenges such as offering financial rewards and benefits (62.7%); implementing a salary scale (47.8%); flexible schedules (31.3%); staff development (29.9%); offering praise, incentives and motivation (19.4%); improving the relationship between nurses and management (19.4%); improving work environment (14.9%); and promotion opportunities (11.9%) [6]. One of the main findings of the study was the mismatch between reported challenges and implemented strategies which will probably lead to further challenges for Nursing Directors in Lebanese hospitals.

There is a need for sound and proven strategies developed by HR managers for recruiting and retaining HR in hospitals. Hospitals need effective Human Resources Management (HRM) to be able to deliver quality and safe care [7]. According to evidence in the literature, effective HRM practices lead to better health and well-being of workers, higher satisfaction, lower absenteeism and turnover, financial advantages (reduced costs, increased productivity), and better quality of care and patient outcomes. Thus effective HRM strategies practiced by HR managers are becoming critical to the success of hospitals [7]. The most prominent challenges to HRM include policies and procedures which hinder the process and delay recruitment and retention; very centralized and fragmented HR management systems; lack of incentives; poor utilization of current staff in addition to absence of proper leadership [8]. In spite of the fact that effective human resources management is essential for the success of organizations, limited knowledge is available about the challenges and the nature of interventions utilized by human resource managers in hospitals including enabling factors and the competences they have or require. In addition, limited knowledge is available on the number, qualifications, experience and competences of existing HR managers in hospitals. This is known in several East Mediterranean countries, and Lebanon is no exception.

To our knowledge, no study has been done in Lebanon and the region to survey HR managers in hospitals about their views on current HR challenges, strategies implemented, and enabling factors including role, education, experience and training.

### Objective

The objective of this study is to assess the HR challenges and strategies as perceived by HR managers in Lebanese hospitals. Specifically, the study is aimed at assessing the perception of HR managers about the challenges they face and the current strategies being adopted. The study also aims at assessing enabling factors including role, education, experience and HR training.

## Methods

A cross-sectional survey design of HR managers (and those who combine their role as HR manager with other duties) working in all Lebanese hospitals was developed. To ensure a balanced design with respect to service and care characteristics, the hospitals were stratified by size (number of beds) into the three categories defined by the Lebanese Ministry of Health as follows: small (≤ 100 beds), medium (101-200 beds) and large (>200 beds).

The survey targeted HR managers (and employees who combine the role of HR manager with other duties) in Lebanese hospitals and was designed based on an extensive literature review and discussions among the research group. The research team used a combination of open- and close-ended questions to allow the HR managers to better document their viewpoints regarding challenges and strategies. Questions included educational background, qualifications, work experience, gender, and age. The survey also included questions about perceived challenges facing the human resources component at hospitals and key strategies to mitigate these challenges. These were open-ended questions so that respondents could freely describe the specific issues pertaining to each question. The survey also addressed other issues such as the categories of human resources with whom HR managers were facing the most challenges in retention, frequency of conducting performance appraisal, trends in assessment of credentialing for medical and nursing staff, existing continuing education or development programs, in addition to the presence of recruitment and retention strategies being utilized by the hospital.

The questionnaire was originally developed in English and then translated to Arabic as it is the primary language of most HR managers in Lebanon. Back translation to English was conducted to validate the Arabic translation. After the questionnaire was finalized, it was pilot tested for both language versions after which minor changes were made to the wording of some questions.

HR managers (and those who combine the role of HR manager with other duties) in all Lebanese hospitals were contacted. Hospitals were asked to forward the survey to individuals in charge of the HR function. When contacted, the hospitals were informed about the purpose and significance of the study. Hospitals were assured that participation was voluntary in addition to the confidentiality and anonymity of their responses. After obtaining informed consent to participate in the study, the questionnaire was provided to HR managers. In some instances, hospitals did not have a designated HR manager, therefore, two or more employees often combined their primary role in the hospital (whether clinical or non-clinical) with the HR management function. In these cases, all employees affiliated with the HR department filled the survey.

All hospitals were sent a fax requesting their participation in the study. A total of 72 hospitals expressed their willingness to participate and 61 hospitals responded to the survey with a total of 97 respondents.

### Data analysis

Data was entered and analyzed using the Statistical Package for Social Sciences (SPSS) 16.0. The quantitative data analysis included uni-variate and bi-variate analysis. The qualitative data analysis comprised thematic analysis of open-ended questions to derive the main challenges and strategies adopted by hospitals as perceived by HR managers. Answers were thematically analyzed and coded. Similar codes were grouped under categories and related categories were then gathered under themes. Strategies were compared against reported challenges to assess whether the adopted strategies can serve to mitigate the impact of the reported challenges. Thematic analysis followed both an inductive and deductive approach whereby some themes were based on a search of the literature (inductive) and others emerged from findings (deductive). The predetermined HR challenges included financial constraints, employee shortages and lack of qualified personnel, migration, poor job satisfaction, recruitment challenges (or lack of such a system), and poor employee retention (incentive programs). As for proposed strategies, the predetermined themes included improving salaries and strengthening incentive plans, enhancing managerial support, developing recruitment and retention strategies, and offering continuing education to staff. Additional challenges and strategies were also derived from the deductive approach.

Analysis of quantitative data included questions on level of education, qualifications in HR management, experience and training in HR management, and plans for continuing education in the realm of HR management, in addition to other information about the hospital where respondents were employed.

## Results

### Characteristics of respondents

When the respondents were asked whether they were in charge of the HR function at the hospital, 68% answered positively, and 42% of those held other jobs in the hospital (mainly administrative positions). The majority of respondents (40.2%) held a bachelors degree (Bachelors of Business Administration (BBA), Bachelors of Arts (BA) or Bachelors of Science (BS), while 26.8% held a masters degree (Masters of Business Administration (MBA), Masters of Arts (MA) or Masters of Science (MS)), and 12.4% a Masters of Public Health (MPH) (See Table 1).

**Table 1:** Qualifications and description of respondents

	N (%)
**Are you the individual in charge of HR department at your hospital?**	
No	31 (31.9%)
Yes	66 (68.1%)
*If yes, do you hold another position as well?*	
*No*	*38 (57.6%)*
*Yes*	*28 (42.4%)*

**Highest level of education**	
High School	2 (2.1%)
BBA/BA/BS	39 (40.2%)
BSN	7 (7.2%)
BT/TS	12 (12.4%)
MBA/MA/MS	26 (26.8%)
MPH	5 (5.2%)
MD	4 (4.1%)
Other	2 (2.0%)

**Qualifications in HRM**	
No	41 (36.4%)
Yes	56 (63.6%)

**Currently pursuing education or training related to HRM**	
No	27 (27.8%)
Yes	70 (72.2%)

**Interested in pursuing education or training related to HRM**	
No	17 (17.5%)
Yes	80 (82.5%)

**Previously attended workshops on HRM over the past 3 years**	
No	46 (47.4%)
Yes	51 (52.6%)

**How long have you been working in this hospital?**	
< 5 years	10 (10.3%)
5.1 - 10 years	8 (8.2%)
10.1 - 15 years	3 (3.1%)
15.1 - 20 years	2 (2.1%)
> 20 years	1 (1%)
Missing	73 (75.3%)
*Mean (Standard Deviation)*	*7.56 (5.57)*

**Have you previously worked in the field of HRM?**	
No	57 (58.8%)
Yes	40 (41.2%)

**Gender**	
Male	25 (25.8%)
Female	72 (74.2%)

**Age**	
Below 30 yrs	19 (19.6%)
Between 30 and 45 yrs	63 (64.9%)
Between 46 and 55 yrs	11 (11.3%)
Over 55 yrs	4 (4.1%)

A total of 63.6% of respondents reported holding some qualifications in HRM and 72.2% reported currently pursuing education or training related to HRM. In addition, 82.5% reported being interested in pursuing education or training related to HR management. However, approximately half the respondents (47.4%) reported not having attended any HRM workshops over the past 3 years.

The question on years of experience had only a 24.7% response rate and thus may not represent the entire sample. Respondents who answered this question had an average experience of 7.56 (± 5.57) years. It is also worth noting that only 41.2% had previously worked in the field of HRM. Most respondents were female (74.2%) and 64.9% were between 30 and 45 years of age.

### HR challenges, strategies and enabling factors

Thematic analysis revealed that challenges varied across respondents and participating hospitals. The most highly reported challenge by respondents was poor employee retention at hospitals (56.7%), particularly for nurses (see Table 2). Lack of qualified personnel (35.1%) ranked second whereby respondents reported that there are few candidates for specific positions in their hospitals. Moreover, some required specialties are not available in universities and schools (e.g. occupational health and safety officers, quality managers, etc.). This may cripple the hospitals' ability to provide quality care, as existing staff members cannot assume these roles. The lack of person/job fit may thus impede the hospitals' ability to provide certain services or meet national hospital accreditation requirements in Lebanon. The lack of a system for performance evaluation (28.9%) also emerged as a major challenge as it has reportedly limited the hospitals' ability to evaluate the competencies and performance of their staff, especially critical staff members. Financial constraints were also reported as a major challenge by 24.7% of respondents, as many staff members may value it more than other forms of incentives. Other less frequently reported challenges included overall employee shortages (10.3%), poor satisfaction (8.3%), competition with other hospitals (particularly governmental hospitals) (8.3%), and limited capacity and authority of the HR department (6.2%). The lack of an HR strategic plan also emerged as a challenge but was only reported by 6.2% of participants (see Table 2).

**Table 2:** Most commonly reported challenges and strategies

	N (%)
**Challenges**	
Poor employee retention	55 (56.7%)
Lack of qualified personnel	34 (35.1%)
Lack of a system for performance evaluation	28 (28.9%)
Challenges in recruitment system	26 (26.8%)
Financial constraints	24 (24.7%)
Employee shortages	10 (10.3%)
Poor satisfaction	8 (8.3%)
Competition by governmental hospitals	8 (8.3%)
No strategic planning	6 (6.2%)
Limited capacity of HR Department	6 (6.2%)

**Strategies**	
Offer continuing education and training for employees	19 (19.6%)
Improve salaries	14 (14.4%)
Develop retention strategies	10 (10.3%)
Develop incentives	8 (8.3%)
Managerial support	7 (7.2%)
Needs assessment of existing challenges	6 (6.2%)
Develop recruitment strategy	5 (5.2%)
Develop an HR strategic plan	5 (5.2%)
Improve overall environment in hospital	5 (5.2%)

**Have strategies been successful? *(based on 68 respondents who reported retention strategies)***	
Yes	54 (79.4%)
No	14 (20.6%)

Respondents were asked to report on some strategies utilized by the hospital to mitigate the impact of the above-reported challenges. Although many respondents reported HRM challenges, a total of 68 respondents (70.1%) reported strategies to mitigate the effect of these challenges. Thematic analysis (reported in Table 2) revealed that the most commonly reported strategy by respondents was offering continuing education and training for employees (19.6%). Hospitals often send some of their employees to workshops or short courses to improve their knowledge on certain aspects of their job. Some hospitals also use credits collected from attending such courses when considering promotion opportunities. Improving salaries ranked second (14.4%) among reported strategies, as many hospitals believe that this may be the only way they can keep their employees. Some hospitals also reported developing retention strategies (10.3%) to better retain their employees; but respondents did not specify exact strategies being utilized. Other hospitals have started developing incentive plans (8.3%), mainly through material rewards, to encourage staff members to remain employed. Managerial support (7.2%) also emerged as an HRM strategy, but was only reported by few respondents. Other strategies included but were not limited to needs assessment of existing challenges (6.2%), developing recruitment strategies (5.2%), developing an HR strategic plan for the hospital (5.2%), and improving overall work environment in the hospital (5.2%) (see Table 2).

It is worth noting that 79.4% of respondents reported that the adopted strategies were successful in improving the status of health workers in surveyed hospitals.

Respondents were asked about enabling factors that foster employee retention, such as conducting performance appraisal and evaluation, in addition to staff retention strategies. When asked about the frequency of conducting performance appraisal, 77.3% reported conducting annual performance appraisal for all of their employees in the hospital (see Table 3). Although conducting performance appraisals is a requirement of the Lebanese hospital accreditation program, our findings imply that not many hospitals recognize its importance for employee retention yet. The remaining hospitals did not report conducting performance appraisals. However, respondents indicated that some specific staff members are often appraised as needed, such as heads of departments, some members of the medical staff, and selected nurses and technicians.

**Table 3:** HR Management trends in participating hospitals

	N (%)
**Does the hospital conduct performance appraisal for all staff members on regular basis?**	
Yes	75 (77.3%)
No	22 (22.7%)

**Does the hospital conduct periodic assessment of credentialing of medical and nursing staff?**	
Yes	61 (62.9%)
No	36 (37.1%)

**Does the hospital have continuing education or career development program for employees?**	
Yes	53 (54.6%)
No	44 (45.4%)

**Does the hospital hold regular training sessions for staff?**	
No	14 (14.4%)
Yes	83 (85.6%)
*In the hospital*	*8 (9.6%)*
*Outside the hospital*	*0 (0.0%)*
*Both*	*74 (89.2%)*
*Missing*	*1 (1.2%)*

**Does the hospital require training on specific skills in HR management?**	
Yes	55 (56.7%)
No	42 (43.3%)

**Does the hospital have a recruitment and retention strategy?**	
Yes	26 (26.8%)
No	71 (73.2%)

Periodic assessment of credentialing for medical and nursing staff was reported by 62.9% of respondents. Furthermore, 54.6% of hospitals reported having a continuing education or career development programs in their hospitals. Most of the HR managers (85.6%) reported that they provided staff with ad-hoc training sessions both in and outside the hospital (89.2%). Moreover, over half the respondents (56.7%) reported a need for training in specific HR skills to help them in their role within this department in their hospital (see Table 3).

Only 26.8% of respondents reported that their hospital has a recruitment and retention strategy. The low percentage on this question may reflect a lack of awareness about the extent to which recruitment and retention strategies are effective HR management tools in Lebanese hospitals (see Table 3).

Respondents were finally asked to select the top three categories of health professionals facing the most challenges at their hospital. The majority of respondents reported that the staff categories facing the most challenges were registered nurses (78.4%), practical nurses (49.5%), and administrative staff (33.0%) (See Table 4). Respondents also reported that they are facing challenges with additional members of the hospital staff, including: housekeeping staff, technicians and casual employees (paid on a daily basis).

**Table 4:** Categories of health professionals facing most challenges

	N (%)
Registered nurse	76 (78.4%)
Practical nurse	48 (49.5%)
Administration	32 (33.0%)
Physicians	18 (18.6%)
Technician	16 (16.5%)
Dieticians	10 (10.3%)
Physical, occupational, or speech therapist	5 (5.2%)
Respiratory therapists	4 (4.1%)
Pharmacists	1 (1.0%)
Unit assistant	1 (1.0%)
*Other*	*18 (18.6%)*

## Discussion

The results of this study indicate that HRM in Lebanese hospitals should be strengthened in order to build capacity to better manage and retain health workers. The findings showed that not all hospitals clearly delineate the departmental responsibilities for its HRM function. This can be demonstrated by the challenges and strategies that emerged from thematic analysis. The most striking observation is the mismatch between challenges and strategies in this study. This finding is similar to an earlier study targeting nursing directors [6], where retention strategies did not always correspond to the reported challenges. However, this does not necessarily imply that the HR managers are not aware of how to address the challenges they reported. On the contrary, it may reflect the limited capacity and authority they have to mitigate challenges that are hindering HR development at their institution. This was actually reported as a challenge by some of the respondents. Another challenge reported by some respondents was the lack of a strategic plan for HR in hospitals. It is worth noting that Lebanese hospitals are currently in the process of preparing for a new national accreditation survey, and the development of a HR strategic plan is a requirement in the Lebanese accreditation standards.

While many themes (related to challenges and strategies) derived from the results of this study correspond well with those derived from the literature, it should be noted that additional challenges and strategies emerged. The additional challenges include: lack of a strategic HR plan, competition with other hospitals (particularly governmental hospitals), limited capacity of the HR department, absenteeism, social constraints, poor communication across departments, hospital location, and lack of trust in hospital administration. As for retention strategies, the additional themes that emerged from the results are: needs assessment for existing challenges; improving work environment; communicating specialties needed at universities and schools; cooperating with other institutions on continuing education for staff members; and cross training to fill vacant positions (for promotion from within hospital). It is clear that many of these additional challenges and reported strategies are specific to the context of Lebanon.

As previously stated, many of the reported strategies deployed by HR managers did not exactly match the reported challenges. However, many of the proposed strategies can remedy to some extent the reported challenges. For instance, the most commonly reported strategy was offering continuing education and training for employees (19.6%). Moreover, 54.6% of respondents reported offering continuing education sessions to staff while 85.6% offer training sessions. Offering continuing education and implementing professional clinical/career ladders have been cited as effective strategies for improving employee retention [9-12] and improving health worker efficiency which is linked to the scaling up of productivity [13]. They are forms of non-financial incentive which allow employees the opportunity to advance in their careers. Further research is needed to asses whether continuing education at Lebanese hospitals is strategic and in line with training needs of staff.

Many respondents revealed that hospitals are engaging in financial incentives in an effort to retain their staff. Despite the attractiveness of financial rewards, it has a limited impact if not combined with improved working conditions, employee motivation and linked to individual performance [14]. It should be noted that only 14.4% of hospitals are engaging in financial incentives, although 24.7% reported having financial constraints that did not allow them to compensate their staff as appropriately as desired. It is also worth noting that some respondents (8.3%) reported that hospitals are beginning to develop incentives without specifying whether they were financial or non-financial. More work is needed to understand the types of incentives used by Lebanese hospitals and their level of success.

Managerial support has been cited as an effective mechanism to improve employee motivation, job satisfaction and retention [15,16]. Managerial support includes but is not limited to coaching and mentoring staff, supporting continuing education pursuits, staffing and scheduling, and mediation between staff and administration, among other responsibilities [15]. Managers also have a leadership role, which is as essential component of employee retention, particularly through encouraging an atmosphere of autonomy and shared governance, in addition to empowerment and group cohesion [16]. Despite the importance of managerial support, only 7.2% of respondents cited it as a retention strategy at their hospital. Furthermore, a mere 10.3% of respondents reported developing retention strategies to counter the HR challenges at their hospitals. However, this does not necessarily imply that hospitals do not recognize the importance of retention strategies. With regard to enabling factors for employee retention, many hospitals reported engaging in performance appraisals (77.3%) and assessment of staff credentials (62.9%). Such practices are now required in the Lebanese hospital accreditation program, and all hospitals are required to comply with standards relating to performance appraisals and credentialing. However, there is a lack of information on the degree of compliance of hospitals with this standard and the types of performance appraisals being used.

Many respondents reported that the strategies adopted by their hospitals were successful in mitigating existing challenges. It is not clear how success was assessed, particularly in that many of the reported strategies did not fully correspond to the reported challenges. This may be an indirect outcome of the qualifications of the respondents and their capacity to fill the position of HR managers. Although some respondents had a masters level degree, the majority reported that it was their working experience that qualified them to fill this role in their hospital. It is worth noting that many of the respondents had dual roles in the hospital which may have affected their perception of the existing challenges and limited their capacity to enforce proper strategies to counter their impact.

## Conclusion

With the upcoming accreditation survey of Lebanese hospitals (2010-2011), there is an opportunity for hospitals to enhance competencies of existing HR managers, and strengthen the HR management component. There is a need to develop a competency framework for the knowledge, skills, attitudes and behavior required for various HR managers. Thorough assessment of what qualifications and experience HR managers have, including all those who work in health care organizations, is required. In this context, there is a need to maintain an adequate number of HR managers in health care organizations with clearly delineated roles, responsibilities and competencies. One of the major findings of this study was that many respondents combine their duties in the HR department with other roles in the hospital. This comes to exemplify the need for a cadre of competent and well-trained HR managers who can fully assume these roles in Lebanese hospitals and work to continuously improve the status of employees at their hospitals. In this context, middle managers (department heads) can play a vital role in HR management and provide supervisory support. These middle managers can participate in selection/recruitment processes of HR; and they can perform supervisory functions related to HR performance management and appraisal. With regard to retention strategies, proper assessment of the impact of current retention strategies in Lebanese hospitals is required. Such information will be crucial to improving HRM practices at the hospital level, and also in providing lessons for peer hospitals, particularly ones that are not currently implementing any retention initiatives.

HRM is a discipline which requires a distinct knowledge base and training. It is not common in certain areas in the health sector at the moment to find professional HR managers, as they are usually promoted from other disciplines. As a result, further education or training is generally required in order to have the necessary competencies to perform well. There is a need to expand HR professional knowledge and competencies for the effective management of human resources in HCOs. There is also a need to increase the pool of competent HR professionals. A new cadre of HR managers will need to be trained and enabled to have real input into operational and strategic decisions about HRM.

Our study findings may apply to other countries in the Eastern Mediterranean Region. Another recent study in nine countries found that health systems suffer from poor HRM, resulting in absence of effective recruitment and retention strategies, poor HR planning, lack of proper performance evaluation mechanisms, and absence of a policy for re-licensing of medical staff [5], and other negative consequences. HRM challenges in HCOs should be valued by policy makers and managers and developing effective HR strategies should become a priority.

## Abbreviations

WHO: World Health Organization; HR: Human Resources; HCO: Health Care Organization; EMR: Eastern Mediterranean Region; HRM: Human Resources Management; SPSS: Statistical Package for Social Sciences; BBA: Bachelors of Business Administration; BA: Bachelors of Arts; MBA: Masters of Business Administration; MA: Masters of Arts; MS: Masters of Science; MPH: Masters of Public Health

## Competing interests

The authors declare that they have no competing interests.

## Authors' contributions

FE made substantial contributions to the conception, design, as well as analysis and interpretation of results. VT substantially assisted with the literature review, data analysis and write-up of the article. DJ made substantial contributions to analysis of data and interpretation of results. All authors read and approved the final manuscript.
